# Autosomal-Dominant Corneal Endothelial Dystrophies CHED1 and PPCD1 Are Allelic Disorders Caused by Non-coding Mutations in the Promoter of *OVOL2*

**DOI:** 10.1016/j.ajhg.2015.11.018

**Published:** 2015-12-31

**Authors:** Alice E. Davidson, Petra Liskova, Cerys J. Evans, Lubica Dudakova, Lenka Nosková, Nikolas Pontikos, Hana Hartmannová, Kateřina Hodaňová, Viktor Stránecký, Zbyněk Kozmík, Hannah J. Levis, Nwamaka Idigo, Noriaki Sasai, Geoffrey J. Maher, James Bellingham, Neyme Veli, Neil D. Ebenezer, Michael E. Cheetham, Julie T. Daniels, Caroline M.H. Thaung, Katerina Jirsova, Vincent Plagnol, Martin Filipec, Stanislav Kmoch, Stephen J. Tuft, Alison J. Hardcastle

**Affiliations:** 1 UCL Institute of Ophthalmology, University College London, London EC1V 9EL, UK; 2 Institute of Inherited Metabolic Disorders, First Faculty of Medicine, Charles University in Prague and General University Hospital in Prague, Ke Karlovu 2, Prague 128 08, Czech Republic; 3 Department of Ophthalmology, First Faculty of Medicine, Charles University in Prague and General University Hospital in Prague, U Nemocnice 2, Prague 128 08, Czech Republic; 4 UCL Genetics Institute, University College London, London WC1E 6BT, UK; 5 Institute of Molecular Genetics, Vídeňská 1083, Prague 142 20, Czech Republic; 6 Weatherall Institute of Molecular Medicine, University of Oxford, John Radcliffe Hospital, Oxford OX3 9DS, UK; 7 Moorfields Eye Hospital, London EC1V 2PD, UK; 8 European Eye Clinic Lexum, Antala Staška 1670/80, Prague 140 00, Czech Republic

## Abstract

Congenital hereditary endothelial dystrophy 1 (CHED1) and posterior polymorphous corneal dystrophy 1 (PPCD1) are autosomal-dominant corneal endothelial dystrophies that have been genetically mapped to overlapping loci on the short arm of chromosome 20. We combined genetic and genomic approaches to identify the cause of disease in extensive pedigrees comprising over 100 affected individuals. After exclusion of pathogenic coding, splice-site, and copy-number variations, a parallel approach using targeted and whole-genome sequencing facilitated the identification of pathogenic variants in a conserved region of the *OVOL2* proximal promoter sequence in the index families (c.−339_361dup for CHED1 and c.−370T>C for PPCD1). Direct sequencing of the *OVOL2* promoter in other unrelated affected individuals identified two additional mutations within the conserved proximal promoter sequence (c.−274T>G and c.−307T>C). *OVOL2* encodes ovo-like zinc finger 2, a C2H2 zinc-finger transcription factor that regulates mesenchymal-to-epithelial transition and acts as a direct transcriptional repressor of the established PPCD-associated gene *ZEB1*. Interestingly, we did not detect *OVOL2* expression in the normal corneal endothelium. Our in vitro data demonstrate that all four mutated *OVOL2* promoters exhibited more transcriptional activity than the corresponding wild-type promoter, and we postulate that the mutations identified create cryptic *cis*-acting regulatory sequence binding sites that drive aberrant *OVOL2* expression during endothelial cell development. Our data establish CHED1 and PPCD1 as allelic conditions and show that CHED1 represents the extreme of what can be considered a disease spectrum. They also implicate transcriptional dysregulation of *OVOL2* as a common cause of dominantly inherited corneal endothelial dystrophies.

## Introduction

Posterior polymorphous corneal dystrophy (PPCD) is an autosomal-dominant disease that primarily affects the corneal endothelium. PPCD is characterized by abnormal corneal endothelial cell morphology and associated Descemet membrane changes, which produce the appearance of vesicular lesions, gray-white opacities, and linear bands on clinical examination of the cornea.^1^ The altered morphology is most likely congenital and either static or slowly progressive throughout life. There could also be iris abnormalities, such as ectropion uveae, corectopia, and adhesions, between the peripheral iris and the posterior surface of the cornea.^2^, ^3^ The disease is associated with a broad phenotypic spectrum ranging from mild symptoms to congenital corneal edema. In a minority of cases, these changes lead to a raised intraocular pressure with secondary glaucoma.^2^, ^4^, ^5^ Up to 33% of PPCD subjects require corneal grafting to treat their corneal edema.^6^, ^7^

PPCD is genetically heterogeneous, and approximately 30% of cases are attributed to haploinsufficiency due to truncating mutations or deletions of the transcription-factor-encoding gene *ZEB1* (MIM: 189909). These mutations cause PPCD3 (MIM: 609141).^8^, ^9^, ^10^ Non-synonymous variants in *COL8A2* (MIM: 120252) are also reportedly associated with PPCD in a minority of cases (PPCD2 [MIM: 609140]).^11^ The majority of genetically unsolved cases are most likely associated with a third PPCD locus (PPCD1 [MIM: 122000]), which was originally mapped to a 30 cM region on the short arm of chromosome 20 (20p).^7^ The PPCD1 locus was subsequently refined in two large families of Czech origin^6^, ^12^ and a large white American family.^5^, ^13^, ^14^

The phenotypes described as PPCD1 share similarities with an autosomal-dominant corneal endothelial dystrophy with a severe phenotype originally termed congenital hereditary corneal edema and subsequently classified as congenital hereditary endothelial dystrophy 1 (CHED1 [MIM: 121700]).^1^, ^4^ However, differences have also been reported. Notably, individuals in the index CHED1-affected family presented with a very severe phenotype often including corneal haze that was evident by 1 year of age and required corneal transplantation.^1^, ^15^ Linkage analysis of this large British kindred mapped the disease to a 2.7 Mb locus on 20p, but the genetic cause has not been determined.^16^ Because PPCD1 and CHED1 map to overlapping loci on 20p, it is speculated that these two disorders could be allelic conditions that are caused by mutations within the same gene but that display variable phenotypic severity. Alternatively, they could be caused by mutations in two different genes at neighboring loci.^1^, ^12^, ^13^, ^14^, ^16^, ^17^

In this article, we describe sequential genetic analyses that excluded copy-number variations (CNVs) and coding and/or splice-site mutations as the cause of CHED1 or PPCD1, and we also identify the cause of disease via a parallel approach of genome sequence analysis. We show that variants in the *OVOL2* promoter sequence cause the spectrum of phenotypes observed in over 100 affected individuals, implicating perturbed transcriptional regulation of *OVOL2* as a major cause of dominant corneal endothelial dystrophies.

## Material and Methods

### Study Subjects and Clinical Examination

The study followed the tenets of the Declaration of Helsinki and was approved by the research ethics committees (RECs) at Moorfields Eye Hospital (REC reference nos. 13/LO/1084 and 09/H0724/25) and the General University Hospital in Prague. After informed consent was obtained, blood samples were donated, and genomic DNA was extracted from lymphocytes or saliva samples via conventional methodologies.

**Figure 1:**
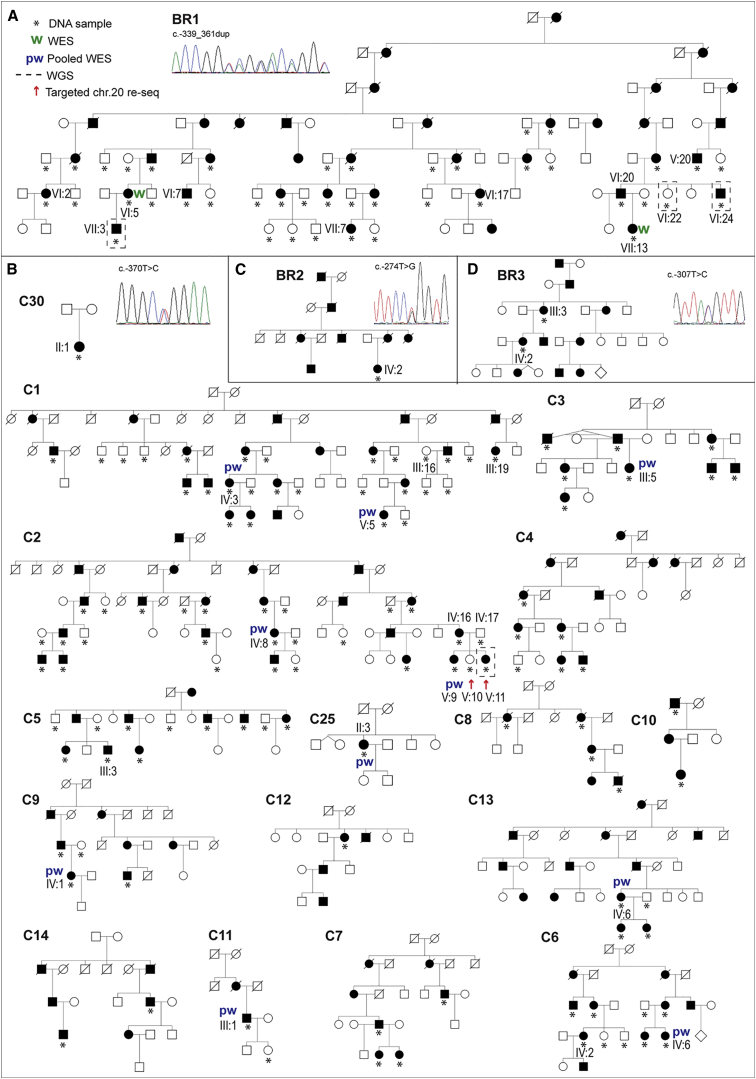
Pedigree Structure of CHED1- and PPCD1-Affected Families with *OVOL2* Promoter Mutations Pedigrees of (A) the index British CHED1-affected family (BR1), (B) 16 PPCD1-affected families (C1–C14, C25, and C30) from the Czech Republic, and further British families (C) BR2 and (D) BR3. For each family (BR1–BR3) or group of families (C1–C14, C25, and C30), a distinct and unique mutation in the *OVOL2* promoter was identified. Sanger sequencing traces representing the four unique heterozygous mutations identified are shown in each respective panel (A–D). Second-degree unaffected relatives are not shown. Mutations are annotated in accordance with GenBank: NM_021220 (Ensembl: ENST00000278780), and +1 represents the A of the ATG translation initiation codon. DNA from individuals, each highlighted with a red arrow, was analyzed by targeted re-sequencing of the disease-associated locus (chr20: 17,335,789–19,665,902). Asterisks indicate that a DNA sample was available for genotyping. Abbreviations are as follows: W, whole-exome sequencing (WES); and pw, pooled WES. Dashed lines indicate that a sample was whole-genome sequenced.

A detailed history was recorded and ophthalmic examination was performed for all available study subjects (highlighted in Figure 1). Specular microscopy (Noncon ROBO Pachy SP-9000, Konan Medical) and spectral-domain optical coherence tomography (SPECTRALIS, Heidelberg Engineering) were performed in select cases.

### Histology

Full-thickness corneas from individuals VII:13 (age 6 years) and VII:7 (age 11 years) from family BR1 and individual III:1 (age 42 years) from family C11 were fixed in 10% neutral-buffered formalin and 10% formalin, respectively. Samples were then processed into paraffin wax, and 4 μm (VII:13 and VII:7) or 6 μm (III:1) sections were cut. Samples from individuals VII:13 and VII:7 (family BR1) were stained with H&E and PAS stains via conventional methods. The sample from individual III:1 from family C11 was stained with H&E only.

### CNV Analysis

Array comparative genomic hybridization (CGH) was used for evaluating DNA CNVs on chromosome 20. DNA from affected individual VII:13 from family BR1 was hybridized (labeled with Cy3) to a dense chromosome 20 array (median probe spacing of 1 per 134 bp, NimbleGen Systems) with a reference DNA sample (labeled with Cy5). The data were visualized with SignalMap software (NimbleGen Systems). DNA from individuals III:19 (affected) and III:16 (unaffected spouse) from family C1 was also previously analyzed via the same methodology.^12^ In addition, data from both SNP and copy-number probes present on Affymetrix Genome-Wide Human SNP Array 6.0 were used for identifying copy-number changes in five members from the Czech familial cohort (individuals IV:16, IV:17, V:9, V:10, and V:11 from family C2) in accordance with previously described methods.^18^, ^19^

### Haplotype Analysis

Haplotype analysis was performed in family BR1 with the aim of refining the previously identified region of linkage.^16^ A combination of SNP and microsatellite markers were genotyped via conventional PCR and direct Sanger sequencing (Figure S1 and Table S1; primer sequences are available upon request). Fluorescently labeled microsatellite markers from the ABI Prism Linkage Mapping Set v.2.5 (Applied Biosystems) were used for genotyping microsatellites. PCR-amplified products were analyzed on an ABI Prism 3100 Genetic Analyzer (Applied Biosystems), and GeneMarker v.1.85 software was used for generating microsatellite genotyping calls.

### Whole-Exome Sequencing

Individuals VII:13 and VI:5 from family BR1 were analyzed by whole-exome sequencing (WES) on a SureSelect XT2 Human All Exon v.4.0 capture kit (Agilent) and a HiSeq 2000 sequencer (Illumina). Reads were aligned to the human reference sequence (UCSC Genome Browser hg19) with Novoalign v.2.05 (Novocraft). ANNOVAR (OpenBioinformatics) was used for annotating SNPs and small indels. ExomeDepth^20^ was used for calling CNVs. Aligned data were visualized with the Integrated Genomics Viewer (Broad institute).

In parallel, pooled DNA samples from ten affected individuals from the Czech familial cohort (highlighted in Figure 1B: IV:3 and V:5 from family C1, IV:8 and V:9 from family C2, III:5 from family C3, IV:6 from family C6, IV:1 from family C9, III:1 from family C11, IV:6 from family C13, and II:3 from family C25) were enriched with SeqCap V3 (NimbleGen) and sequenced on an Illumina HiSeq 1500 system at the University Hospital in Motol (Prague). The resulting FASTQ files were aligned to the human reference genome (UCSC Genome Browser hg19) with Novoalign. After genome alignment, conversion of SAM format to BAM and duplicate removal were performed with Picard Tools (v.1.129). The Genome Analysis Toolkit (GATK v.3.3) was used for local realignment around indels, base recalibration, and variant recalibration and genotyping. Variants were annotated with SnpEff^21^ and GEMINI,^22^ and CNVs were identified from exome read counts with CoNIFER (v.0.2.2.).^22^

### Targeted Capture and Sequencing of the PPCD1 Locus

A custom Sequence Capture 385K Human Array targeting the PPCD1 linkage region on chromosome 20 between D20S48 and D20S107^6^ was designed and manufactured by Roche NimbleGen. DNA enrichment from approximately 20 μg of DNA from affected individual V:11 and unaffected sibling V:10 from family C2 was performed at NimbleGen Customer Service. Sequencing was performed on a Roche 454 FLX instrument at the Institute of Molecular Genetics in Prague according to the manufacturer’s protocol. The reads were processed and aligned to the human reference genome (UCSC Genome Browser hg19) with the Burrows-Wheeler Aligner Smith-Waterman alignment (BWA-SW).^23^ Putative DNA variants were detected with SAMtools (v.0.1.12). Unique DNA variants were identified with SIFT 4.0.3^24^ and SeattleSeq.

### Whole-Genome Sequencing

DNA samples from affected individuals VII:3 and VI:24 and unaffected individual VI:22 (age 44 years) from family BR1 were analyzed by whole-genome sequencing (WGS) with a TruSeq Nano DNA Library Preparation Kit (Illumina) and a HiSeq X Ten sequencer (Illumina). Generated reads were aligned to the human reference genome (UCSC Genome Browser hg38) with Novoalign. Variant calling was performed with GATK (variants were first called per individual sample with the GATK HaplotypeCaller module, and then calls were improved with joint calling in the GenotypeGVCFs module). Both coding and non-coding variants were annotated with the Variant Effect Predictor (VEP). All variants were annotated with 1000 Genomes allele frequencies. Additionally, coding variants were annotated with allele frequencies from the Exome Aggregation Consortium (ExAC) Browser. Variants were further annotated with Combined Annotation Dependent Depletion (CADD), Combined Annotation Scoring Tool (CAROL), and Consensus Deleteriousness (Condel) consequence scores for assessment of their functional impact. Subsequently, affected individual V:11 from family C2 was also analyzed by WGS via the same methodology.

### *OVOL2* Sanger Sequencing

Members of families BR2 and BR3 were screened for *OVOL2* mutations in the coding sequence, splice sites, and 1.8 kb of the promoter region by direct Sanger sequencing from PCR amplimers (primer sequences are available upon request) via conventional methodologies. Segregation analysis of *OVOL2* variants was also performed by PCR amplification and direct Sanger sequencing in families BR1, C1–C11, C13, C14, and BR3. Two hundred and nine ethnically matched white British control DNA samples were also screened for variants in the *OVOL2* promoter region, encompassing all mutations identified in this study. In addition, Czech control DNA samples were screened by PCR amplification and restriction fragment digest using restriction enzyme Cfr13I (Fermentas) as a test for the presence or absence of the c.−370T>C *OVOL2* variant found in Czech PPCD1 individuals (primers and reaction conditions are available upon request). All *OVOL2* variants are annotated in accordance with GenBank: NM_021220, and +1 represents the start of translation.

### In Silico Analysis of Promoter Variants

Bioinformatic analyses of regulatory motifs and potential transcription factor binding sites were compared between wild-type and mutated promoter sequences with the programs Alibaba 2.1 and MatInspector.^25^

### Analysis of RNA-Sequencing Data

Human corneal endothelial RNA-sequencing (RNA-seq) reads for three adult and two fetal (16- to 18-week-old) samples (study SRP01140 from ArrayExpress) were aligned to the human reference genome (NCBI Genome build GRCh38; GCA_000001405.15_GRCh38 without alternate contigs; see Web Resources) with STAR v.2.5.0. Duplicate reads were marked with Picard MarkDuplicates v.1.100. Raw read counts, excluding duplicate reads, were generated with DEXSeq python scripts (dexseq_count.py). The resulting counts were normalized according to the length of each feature (estimated with the Rsubread package) and a library-size factor as estimated by the DEXSeq tool. Gene annotations were based on Ensembl transcripts and downloaded from the Ensembl page (GRCh38.82).

### Cell Culture, RNA Extraction, and RT-PCR

Whole human corneal tissue was donated after enucleation surgery due to posterior segment melanoma. Corneal endothelial tissue was also donated by individuals who had Descemet membrane endothelial keratoplasty (DMEK) surgery for Fuchs endothelial corneal dystrophy. Primary endothelial cells were expanded and cultured in accordance with previously described methods.^26^ An immortalized cell line of human corneal endothelial origin, B4G12, was cultured according to published protocols.^27^ Human stromal keratocytes were isolated from surgically removed central graft tissue from a donor without any history of ocular disease as described previously.^28^ Fibroblasts were derived from expanding the cells in media supplemented with serum.^28^ Human corneoscleral rims stored in Optisol (Chiron Ophthalmics) from donors who provided research consent were obtained from the Moorfields Eye Hospital tissue bank, and limbal epithelial stem cells were isolated and cultured as previously described.^29^ HEK293 cells were cultured with standard reagents and conditions.

Total RNA was extracted from cells or tissue with an RNeasy Extraction Kit (QIAGEN) according to the manufacturer’s protocol. cDNA was reverse transcribed with a Tetro cDNA Synthesis Kit (BIOLINE) and an oligo (dT)_18_ primer mix. For RT-PCR reactions, *OVOL2* was amplified with intron-spanning primers 5′-CTCGCGATTTAAGGCATAGG-3′ and 5′-ACAGCTGTGAACCACCGAGT-3′ from exons 1–3. Beta-actin was also amplified as a positive control with intron-spanning primers 5′-CTGGGACGACATGGAGAAAA-3′ (forward) and 5′-AAGGAAGGCTGGAAGAGTGC-3′ (reverse).

### Luciferase Assay

Primers were designed to amplify a 1,824 bp fragment of the *OVOL2* promoter (chr20: 18,057,635–18,059,458) from control human genomic DNA. The fragment was cloned into pGEM-T Easy (Promega) and subsequently sub-cloned, with MluI and BglII restriction sites, into the promoter-less firefly luciferase reporter vector, pGL3-Basic (Promega), according to standard protocols for the generation of pGL3-*OVOL2*. Variants of interest were introduced into the *OVOL2* promoter sequence by site-directed mutagenesis with a Q5 Site-Directed Mutagenesis Kit (New England Biolabs) in accordance with the manufacturer’s protocol. All constructs generated were Sanger sequenced for ensuring fidelity and orientation (primers are available upon request).

HEK293 cells were seeded in 96-well plates and transfected at 80% confluency with TransIT-LT1 Transfection Reagent (Mirus). Each well was transfected with a total of 100 ng of plasmid DNA, including 90 ng of pGL3-*OVOL2* or pGL3-Control (the SV40-enhancer- and promoter-containing positive control construct), and 10 ng of the internal control pRL-CMV, a cytomegalovirus (CMV)-promoter-driven *Renilla* luciferase reporter vector (Promega). Twenty-four hours after transfection, luciferase activity was measured on an Orion L Microplate Luminometer (Titertek Berthol) with a dual-luciferase reporter assay system (Dual-Glo Luciferase Assay System, Promega) in accordance with the manufacturer’s protocol.

## Results

### Clinical Characterization of CHED1 and PPCD1

**Figure 2:**
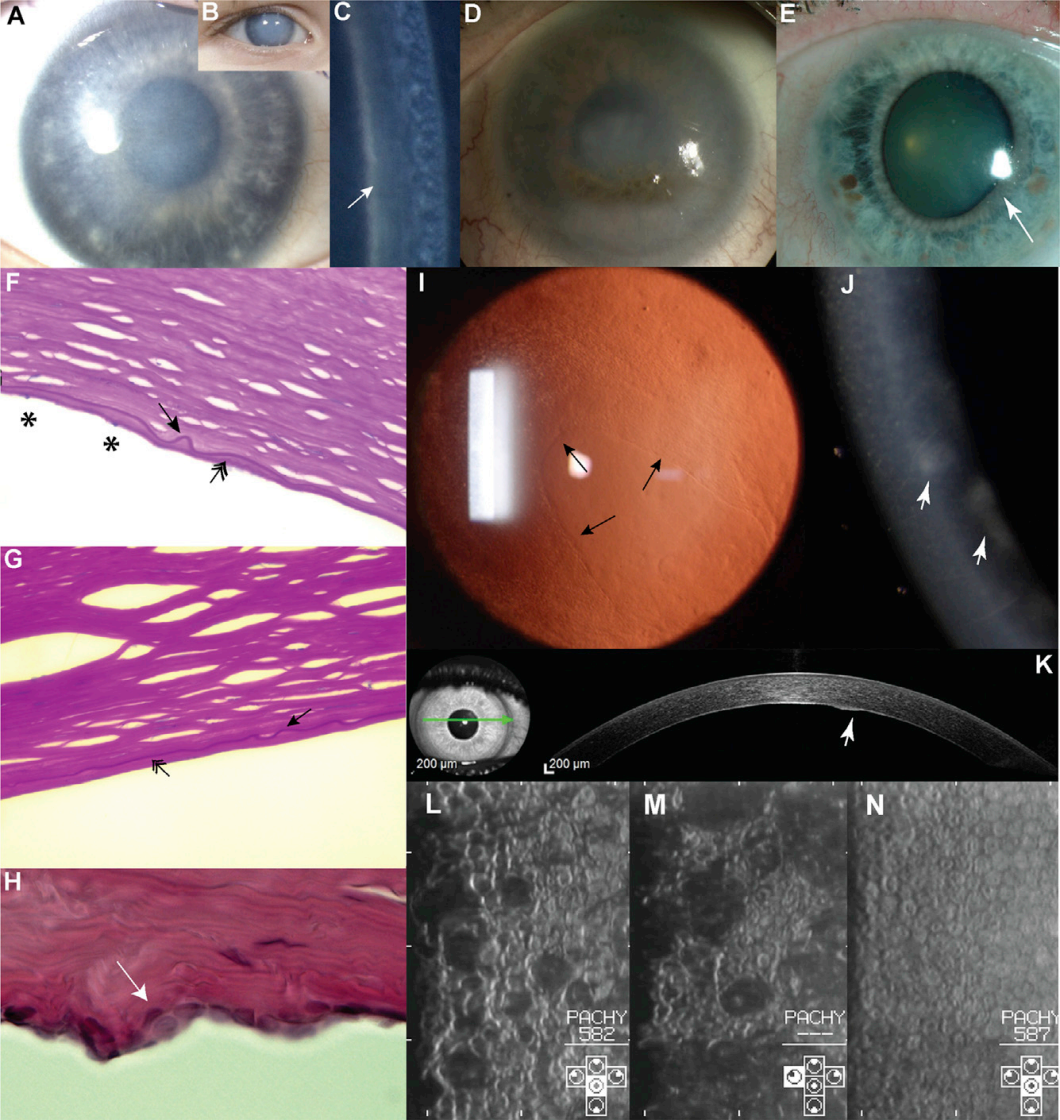
Spectrum of Corneal Disease Associated with *OVOL2* Mutations (A–C) Individual VII:13 from family BR1 at age 3 years. The right (A) and left (B) corneas have a hazy appearance on direct illumination and an increased thickness from diffuse corneal edema. (C) A prominent Descemet membrane can be seen with narrow-beam illumination (arrow). (D) Individual VI:2 from family BR1 at age 52 years (without surgery) shows secondary lipoidal degeneration. (E) Individual III:3 from family BR3 shows a clear cornea but a distorted iris (arrow) secondary to the presence of a peripheral area of iris-to-corneal adhesion (not shown). (F–H) Histological specimens. (F) Individuals VII:13 (age 6 years) and (G) VII:7 (age 11 years) from family BR1. Both individuals have a thin and irregular Descemet membrane (arrow), reduced endothelial cell count (asterisks), and accumulation of material posterior to the Descemet membrane, possibly reflecting mild retrocorneal fibrosis (double-headed arrow). (H) Individual III:1 from family C11 (42 years) shows focal multilayering of endothelial cells (arrow) and undulation of the posterior corneal surface. (I) Retroillumination image of the cornea of individual II:1 (age 29 years) from family C30 shows a mild presentation. An island of normally appearing endothelial cells is surrounded by abnormal cells (arrows highlight the boundary of cells with a normal appearance). (J and K) Individual III:5 (age 33 years) from family C3. (J) A narrow-beam section of the right cornea demonstrates a slight corneal haze and gray focal areas at the level of the Descemet membrane and endothelium (arrowhead). (K) An ocular-coherence-tomography cross-section of the cornea demonstrates a raised lesion on the posterior corneal surface (arrowhead). (L and M) Corneal endothelial specular images from individual V:11 (age 13 years) from family C2. Note the variation in the individual size and shape of the endothelial cells. The dark areas presumably correspond to elevated regions. (N) For comparison, the corneal endothelial specular image of a 25-year-old unaffected sister of individual V:11 (V:10 from family C2) shows a normal appearance of cells with a uniform size and shape.

The clinical and histological features of disease in family BR1 (Figure 1A) were first reported in 1969 as autosomal-dominant congenital endothelial corneal dystrophy,^4^ and further comments on selected individuals were provided in 1987.^15^ The condition was subsequently mapped to a 2.7 Mb region on 20p between markers D20S48 and D20S471 in 1995.^16^ Here, we re-visit this index family (BR1), extend the pedigree, and provide a summary of the phenotype (Figures 1A, 2A–2D, 2F, and 2G). The pedigree now comprises 36 affected individuals spanning seven generations (Figure 1A). The disease appears to be fully penetrant and has no reported systemic associations. Affected individuals typically show symptoms of epiphora and photophobia from birth, and corneal haze is usually noted by 1 year of age. Raised intraocular pressure or iris abnormality was not present in individuals prior to corneal transplantation (Figures 2A–2D). Current data are available on 16 affected individuals from family BR1. All have received at least one corneal graft, or keratoplasty (multiple transplants were often needed after graft failure), as well as additional surgeries for secondary glaucoma. Three have also had a keratoprosthesis (e.g., Boston keratoprosthesis), and three have had an eye enucleated. Histological examination of full-thickness corneas for two previously unreported affected individuals, VII:13 (age 6 years) and VII:7 (age 11 years), revealed a thin and irregular Descemet membrane, reduced endothelial cell count, and accumulation of material posterior to the Descemet membrane, possibly reflecting mild retrocorneal fibrosis (Figures 2F and 2G).

Previous haplotype, geographic, and statistical analyses demonstrated that unknowingly related PPCD1-affected families from the southwestern region of the Czech Republic (around the town of Klatovy) harbor an undiscovered mutation on 20p11.23 and that this mutation most likely arose in a common ancestor originating more than 64 generations ago.^12^ The number of PPCD1-affected pedigrees originating from this region of the Czech Republic (these families constitute those described here) has been extended and is represented in Figure 1B. Sixteen pedigrees with over 100 affected individuals were identified. PPCD1 in this cohort is fully penetrant and has no reported systemic associations. Affected members of these families present with irregularities of the otherwise smooth posterior corneal surface and often have focal opacities and geographic lesions of abnormally appearing cells (Figures 2I–2K). The corneal endothelium exhibits occasional multi-layering^30^ (Figure 2H). Microscopic visualization of the specular reflection from the posterior corneal surface further documents abnormal endothelial cell morphology and irregularities of the posterior corneal surface (Figures 2L and 2M). One-third of these subjects have had a keratoplasty in at least one eye. Additionally, approximately 30%, including some individuals who had not had corneal transplantation, show secondary glaucoma. The phenotype, including the necessity for keratoplasty and occurrence of secondary glaucoma for some affected individuals, has been described previously.^6^, ^31^ In contrast to family BR1, none of the affected members had corneal edema present at birth; the earliest manifestation was in two 5-year-old children (individuals III:3 C5 and IV:2 from C6), which is exceptionally early in this cohort. Out of 75 genotyped Czech PPCD1 individuals, only six underwent keratoplasty before the age of 18 years.

To identify the genetic cause of corneal endothelial disease(s) in the large British kindred and the Czech families, we undertook a sequential genetic approach with parallel and integrated investigations.

### Genetic Analysis of Family BR1

**Figure 3:**
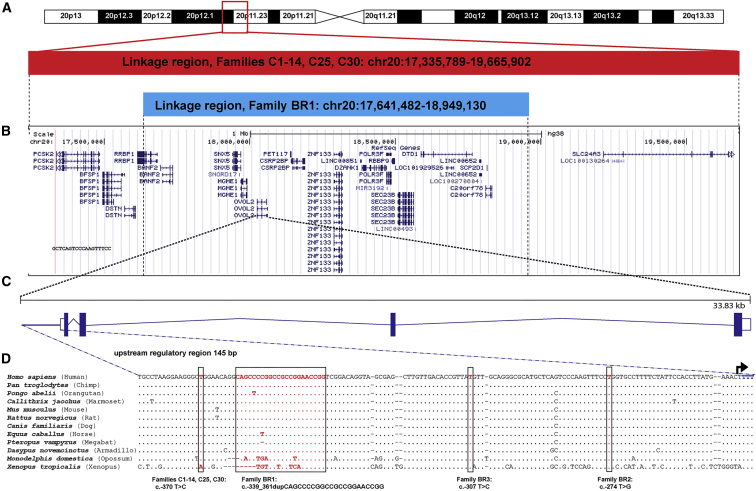
Schematic Representation of the Overlapping Loci, Conservation of the *OVOL2* Promoter, and Relative Position of the Four Mutations (A) Overlapping disease-associated loci on 20p for the Czech familial cohort^12^ and the refined region in family BR1 (Figure S1). (B) Annotated transcripts within the linked regions. (C) Schematic illustration of *OVOL2*, which comprises four exons and encodes a 275 aa protein. (D) ClustalW2 multiple alignment of the promoter region of 11 *OVOL2* orthologs indicates the position of the four mutations identified. The following orthologs were used for the alignment: *Homo sapiens* (Ensembl: ENSG00000125850), *Pan troglodytes* (Ensembl: ENSPTRG00000013280), *Pongo abelii* (Ensembl: ENSPPYG00000010732), *Callithrix jacchus* (Ensembl: ENSCJAG00000005049), *Mus musculus* (Ensembl: ENSMUSG00000037279), *Rattus norvegicus* (Ensembl: ENSRNOG00000006850), *Canis familiaris* (Ensembl: ENSCAFG00000005453), *Equus caballus* (Ensembl: ENSECAG00000007899), *Pteropus vampyrus* (Ensembl: ENSPVAG00000001140), *Dasypus novemcinctus* (Ensembl: ENSDNOG00000025067), *Monodelphis domestica* (Ensembl: ENSMODG00000005504), and *Xenopus tropicalis* (Ensembl: ENSXETG00000024897). The alignment illustrates the conservation of a 145 bp promoter region in which the four disease-associated mutations were identified (mutated base pairs are highlighted in red and boxed). The transcription start site is indicated with an arrow. Mutations are annotated in accordance with the *OVOL2* cDNA sequence (GenBank: NM_021220; Ensembl: ENST00000278780), and +1 represents the A of the ATG translation initiation codon.

To refine the locus in the extended pedigree, we genotyped microsatellite markers and SNPs in eight affected individuals from different branches of the family (individuals V:20, VI:2, VI:7, VI:17, VI:24, VII:3, VII:7, and VII:13; Figure 1A). The disease interval was refined from a 2.7 Mb region to a 1.3 Mb region at chr20: 17,641,482–18,949,130, which encompasses 46 annotated transcripts (Table S1 and Figure 3).

We then used a dense chromosome-20-specific array to perform CGH on an affected individual (VII:13) to evaluate potential CNVs at this locus. This approach did not identify any potentially disease-associated CNVs (data not shown). Next, we performed WES for two affected individuals, VII:13 and VI:5 (Figure 1A). Given the apparent autosomal-dominant inheritance pattern of disease within the family, we assumed that the causal disease variant would be present in the heterozygous state, shared between the two distantly related affected individuals, and given the rarity of the condition, likely to have a minor allele frequency (MAF) < 0.5% in the ExAC database. Filtering the WES data according to these assumptions left only one rare non-synonymous heterozygous variant that was shared between the two affected individuals (Table S2). The identified missense variant, c.1540A>C (p.Ile514Leu), is on chromosome 20 in *DZANK1* (GenBank: NM_001099407), which encodes a predicted transcription factor of undetermined function. This variant is located within our refined CHED1 locus and was experimentally verified to segregate with disease in the extended pedigree by Sanger sequencing. The variant was identified in 5/66,682 ethnically matched European alleles, and in silico analysis of this variant predicted that it is likely to be a benign polymorphism (Table S2). We further analyzed the WES data with ExomeDepth to identify any potentially causative exonic CNVs that are shared between the two affected individuals and that might have been missed by dense array CGH analysis specific to chromosome 20.^20^ No potentially deleterious small indels or CNVs were identified within genes in the disease interval on chromosome 20 or in any known genes related to corneal dystrophies.

Given that no variants likely to be associated with disease were identified by a CGH or WES approach, we next performed WGS on DNA samples from two affected individuals (VII:3 and VI:24) and an unaffected first-degree relative (VI:22, age 44 years), for filtering purposes, to explore the possibility that the causal mutation might be in an intragenic or regulatory region within the refined locus in chr20: 17,641,482–18,949,130 (Figures 1A and 3). First, we filtered our WGS datasets to exclude all common variants that have a MAF > 0.5% within the linked region and that are present in the 1000 Genomes (Ensembl API version 78), ExAC, UK10K, and GoNL datasets, as well as a University College London (UCL) cohort comprising a further 100 WGS datasets (UCL-WGS). We then removed all variants present in unaffected individual VI:22 or in a further eight WGS unrelated samples that were sequenced via the same methods at the same time for other purposes. Using this approach, we found that both affected individuals share 19 variants in the refined locus (chr20: 17,641,482–18,949,130) in the heterozygous state (Table 1). These include the *DZANK1* variant (identified previously via WES), 11 intergenic variants of unknown significance, five deep intronic variants, a variant located within an annotated non-protein-coding transcript (*C20orf78*), and a unique variant within the defined promoter region of *OVOL2*: g.18057974_18057995dup (c.−339_361dup) (Table 1). This heterozygous duplication, located within the *OVOL2* promoter, fully segregates with disease in the family (21 affected and 12 unaffected first-degree relatives; Figure 1A) and is absent in 209 ethnically matched white British control samples (Sanger sequencing).

### Genetic Analysis of Czech PPCD1-Affected Families

In parallel, we independently investigated the genetic cause of disease in 16 Czech PPCD1-affected families (Figure 1B), 12 of which were previously shown to share an ancestral PPCD1 disease haplotype at chr20: 17,335,789–19,665,902 (Figure 3A).^12^ DNA from an affected individual was analyzed by array CGH using a dense chromosome-20-specific array. Furthermore, DNA from three additional affected individuals was analyzed with an Affymetrix Genome-Wide Human SNP Array 6.0. Neither independent method revealed any CNVs within the disease locus.^12^

Next, we performed WES by using pooled DNA from ten affected individuals from the Czech familial cohort (highlighted in Figure 1B: IV:3 and V:5 from family C1, IV:8 and V:9 from family C2, III:5 from family C3, IV:6 from family C6, IV:1 from family C9, III:1 from family C11, IV:6 from family C13, and II:3 from family C25). Given the apparent autosomal-dominant mode of inheritance, we filtered the data under the assumption that the causal disease variant would be present in the heterozygous state in all ten affected individuals and that the casual variant would have a MAF < 0.5% in the ExAC Browser. On the basis of these assumptions, no rare non-synonymous exonic heterozygous variants were found to be shared among the analyzed affected individuals.

Given the possibility that a shared variant could be missed by the WES pooling strategy or that the variant might lie within a non-coding or regulatory region, we employed a targeted resequencing approach with a custom sequence capture designed to target the mapped interval (chr20: 17,335,789–19,665,902) in the Czech families (Figure 3). DNA samples for one affected individual (V:11) and one unaffected individual (V:10, age 30 years) from family C2 were selected for targeted re-sequencing. We filtered annotated sequence data to exclude (1) variants present in the unaffected individual and (2) variants with a MAF > 0.5% in the 1000 Genomes, ExAC, UK10K, and GoNL datasets, as well as an additional 108 UCL-WGS samples. Because of relatively poor coverage in some of the target region, we also performed WGS for one affected DNA sample (individual V:11 from family C2) to ensure that all variants were represented in these datasets. Next, we refined the list of variants to those present on the affected haplotype by directly sequencing each variant in the married-in unaffected father (individual IV:17 from family C2). This reduced the number of affected haplotype variants within the PPCD1 locus (chr20: 17,335,789–19,665,902) to 18, including 12 intergenic variants of unknown significance, five deep intronic variants, and a unique variant within the defined promoter region of *OVOL2*: g.18058004A>G (c.−370T>C) (Table 2). The c.−370T>C variant segregates with disease in all 16 pedigrees shown in Figure 1B (it is present in 75 affected and absent in 21 unaffected first-degree relatives) and is absent in 216 ethnically matched Czech control samples. Strikingly, this *OVOL2* variant is located 9 bp upstream of the duplication identified in the *OVOL2* promoter in family BR1 (Figure 3). Notably, no variants likely to be associated with disease were identified in *DZANK1.*

Using the VEP, we analyzed all unique variants in CHED1 and PPCD1 (Tables 1 and 2) to determine whether any are located in potentially functional regulatory regions. Only two variants were found to be situated within regulatory regions, and both are located within the *OVOL2* promoter (Tables 1 and 2). No other unique variants were found in predicted regulatory regions.

### Screening *OVOL2* as a Candidate Gene in Genetically Unsolved PPCD Cases

To determine whether *OVOL2* mutations could account for disease in further cases of PPCD, we screened eight British and Czech probands with genetically unsolved PPCD by bi-directional Sanger sequencing of the entire coding region, intron-exon boundaries, and 1.8 kb of the promoter region. No mutations were identified within the coding region or exon-intron splice sites of the gene; however, we identified two unique variants in this cohort within the highly conserved proximal promoter region of *OVOL2*. These variants were found to be located in proximity to the disease-associated variants identified by WGS in family BR1 and the founder Czech families (Figure 3D).

One individual (IV:2) from family BR2 (Figure 1C) was available for clinical examination and genetic testing. She reported an autosomal-dominant family history (Figure 1C) and has had poor vision from corneal opacity since childhood. Her phenotype could not be re-assessed because her right eye had had multiple corneal graft procedures and glaucoma drainage surgery and because the left eye had been enucleated. A heterozygous variant, c.−274T>G (g.18057908A>C), located within the *OVOL2* promoter was identified by direct Sanger sequencing (Figure 1C and Figure 3D). This variant is absent in 209 ethnically matched control samples (direct Sanger sequencing) and the 1000 Genomes and UCL WGS datasets.

Two individuals (III:3 and IV:2) from family BR3 (Figure 1D) were available for clinical examination and genetic evaluation. They have an inherited autosomal-dominant form of PPCD, which is less severe than that in families BR1 and BR2. Both individuals showed mild vision loss with a best-corrected LogMar visual acuity of 0.2, and neither has glaucoma or has required corneal surgery. The individuals showed bilateral opacities at the level of the Descemet membrane and an irregular posterior corneal surface with peripheral adhesions between the iris and cornea (one individual also showed a distorted iris in one eye) (Figure 2E). This phenotype is more similar to that in the Czech families (C1–C14, C25, and C30) than that in families BR1 and BR2. Direct Sanger sequencing of the *OVOL2* promoter in individual IV:2 (Figure 1D) identified a unique heterozygous variant, c.−307T>C (g.18,057,941A>G), within the highly conserved region (Figure 3D). This variant was also found in the heterozygous state in her affected mother (individual III:3) but is absent from 209 ethically matched control samples (Sanger sequencing) and the 1000 Genomes and UCL-WGS datasets.

### *OVOL2* as a Candidate Gene for PPCD1 and/or CHED1

*OVOL2* encodes ovo-like zinc finger 2, a C2H2 zinc-finger transcription factor, and we considered it to be an excellent candidate gene for PPCD1 and/or CHED1. The established PPCD-associated gene *ZEB1* encodes a zinc-finger transcription factor that drives epithelial-to-mesenchymal transition (EMT) via repression of genes, including *CDH1* (E-cadherin [MIM: 192090]).^32^ *OVOL2* induces mesenchymal-to-epithelial transition (MET) via direct repression of *ZEB1* expression, and the two genes operate in a regulatory feedback loop that controls cellular plasticity.^33^

We sought to identify where *OVOL2* is expressed in the adult human cornea by RT-PCR using a variety of corneal tissues and cultured cells. *OVOL2* expression was detected in full-thickness corneal tissue, but we were unable to detect expression in adult human endothelial cells derived directly from control tissue, cultured cells expanded from adult corneal endothelial tissue, or an immortalized cell line of human corneal endothelial origin (Figure S1). Interestingly, interrogation of RNA-seq data for human corneal endothelial samples also confirmed that *OVOL2* was not expressed in fetal (16–18 weeks of gestation) or adult corneal endothelium.^34^ These are important data because they demonstrate that *OVOL2* is not normally expressed in the tissue of interest. Furthermore, *OVOL2* expression was absent from cultured stromal fibroblasts (Figure S1). *OVOL2* expression has previously been detected in epithelial tissues, including skin, kidney, and the germinal epithelium of the testis.^35^ Similarly, we detected *OVOL2* expression in a human corneal epithelial culture derived from limbal epithelial stem cells and in a spontaneously immortalized human corneal epithelial cell line with progenitor-like characteristics^36^ (Figure S1). These data support the hypothesis that mutations in the *OVOL2* promoter are likely to exert a gain-of-function effect as a result of inappropriate ectopic expression in the developing or adult corneal endothelium.

### Disease-Associated Mutations Alter Activity of the *OVOL2* Promoter

Given that PPCD3 is caused by presumed haploinsufficiency of *ZEB1*,^10^, ^37^ we hypothesized that the mutations identified in the *OVOL2* promoter might cause disease through dysregulated *OVOL2* expression, potentially by a mechanism leading to overexpression of *OVOL2* and reduced *ZEB1* expression.^33^, ^38^

**Figure 4:**
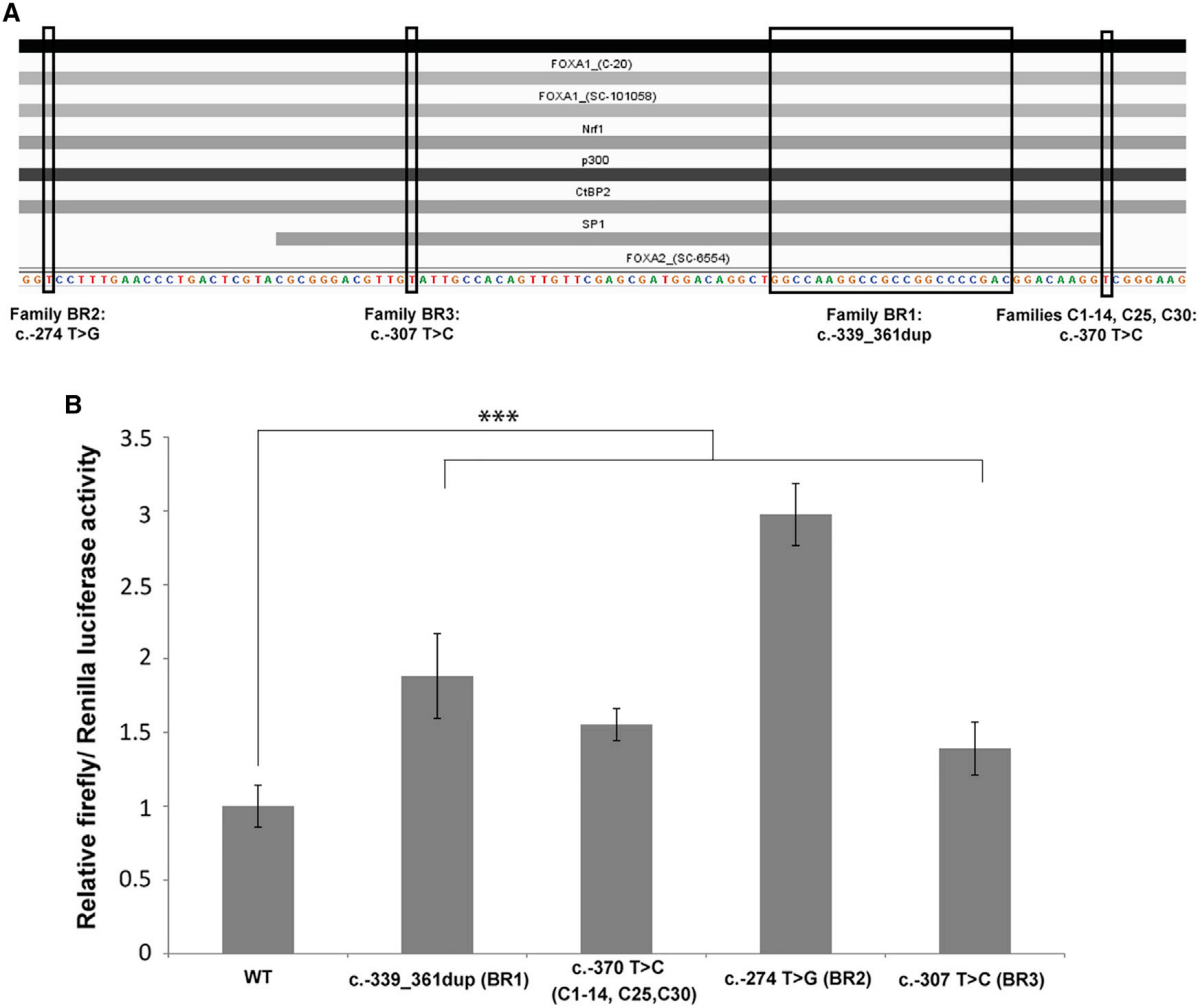
Functional Analysis of the *OVOL2* Promoter (A) *OVOL2* mutations are located within a transcriptionally active region of the promoter. Interrogation of publically available ChIP-seq data released as part of the ENCODE project demonstrates that multiple transcription factors, including FOXA1, FOXA2, NRF1, SP1, CTBP2, and EP300, bind the region encompassing all *OVOL2* disease-associated mutations. (B) Mutations in the *OVOL2* promoter cause an increase in gene expression in vitro. A dual-luciferase reporter assay was used for assessing the impact of disease-associated mutations on the activity of the *OVOL2* promoter. HEK293 cells were co-transfected with pRL-CMV (*Renilla* luciferase) and pGL3-Basic (firefly luciferase) containing 1,824 bp of the wild-type or respective mutant *OVOL2* promoter sequence. The ratio of firefly to *Renilla* luciferase activity was calculated for all samples. Wild-type data were normalized to 1, and the relative luciferase activity in all other samples is expressed in relation to the wild-type data. All mutations investigated significantly increased the relative luciferase activity. Data represent a minimum of three independent experiments with triplicate measurements. Error bars represent ± 1 SD. p values were calculated by one-way ANOVA (^∗∗∗^p ≤ 0.001).

The four mutations cluster within an evolutionary conserved region of the *OVOL2* proximal promoter region (between −370 and −274 bp upstream of the translation start site: Figure 3D). Typically, such regulatory regions upstream of core promoter sequences contain multiple transcription-factor-specific binding motifs that cooperatively stimulate transcriptional activity of a given gene.^39^ Initial interrogation of chromatin immunoprecipitation sequencing (ChIP-seq) data published as part of the ENCODE dataset revealed that it is a transcriptionally active region with binding sites for multiple transcription factors, including FOXA1, FOXA2, NRF1, SP1, CTBP2, and EP300 (Figure 4A). We therefore employed the in silico prediction programs Alibaba 2.1 and MatInspector to test for potential altered transcription factor binding when the four mutations are introduced; compared to the wild-type, all four variants independently resulted in altered predicted transcription factor binding sites (Table 3). Interestingly, interrogation of RNA-seq data for human adult and fetal corneal endothelial cells revealed that the majority of these transcription factors were expressed in the tissue of interest and, therefore, could represent biologically relevant transcription factors.

Next, we experimentally tested the sequence variants in vitro for altered promoter activity by performing a dual luciferase reporter assay. It has been previously established that 1.8 kb of the murine *Ovol2* promoter is sufficient to drive expression of luciferase in HEK293T cells.^40^ Given that the respective syntenic regions share a high level of homology (Figure 3D), we cloned an equivalent 1,824 bp fragment of wild-type sequence and the four respective mutant *OVOL2* promoter sequences into the promoterless firefly luciferase expression vector pGL3-Basic.

Our working hypothesis is that dysregulation of *OVOL2* occurs during development. Given that the corneal endothelium is derived from the neural crest during embryonic development, we wanted to use an appropriate cell line for the luciferase assay. Despite their name, HEK293 cells most likely originate from an embryonic adrenal gland precursor structure^41^ and have been shown to express a number of neuronal markers.^42^ This suggests that they derive from adrenal medulla progenitors, which are themselves derived from the ectoderm of the neural crest. Furthermore, we determined that *OVOL2* is expressed in the HEK293 cell line, suggesting that appropriate regulatory transcription factors might also be expressed (Figure S1). HEK293 cells were co-transfected with each *OVOL2* promoter construct in combination with pRL-CMV for normalization. The 1,824 bp wild-type sequence was sufficient to drive expression of firefly luciferase, and each of the four respective mutants was independently found to significantly increase promoter activity in vitro (p ≤ 0.001) (Figure 4B).

## Discussion

The genetic cause of PPCD1 and CHED1 has proved elusive because conventional gene-screening strategies have failed to investigate potential promoter variants as the cause of corneal endothelial dystrophies linked to chromosomal region 20p11.23. In the promoter of *OVOL2*, we have identified four mutations that segregate with disease in over 100 affected individuals. Our data demonstrate that CHED1 and PPCD1 are allelic conditions representing extremes of disease severity, and we support future use of the nomenclature PPCD1 to represent this spectrum of corneal endothelial dystrophies.^1^ The more severely affected individuals (as described for family BR1) have been symptomatic since birth and displayed corneal haze by 1 year of age. The majority have now had surgery in the form of corneal transplantation, which has often had a poor outcome requiring multiple grafts and caused a tendency for visual loss from corneal opacity and secondary glaucoma. In many cases, visual deprivation in childhood has led to amblyopia and nystagmus. A similarly severe phenotype (early visual loss, keratoplasty, and glaucoma) has also been reported in a further family linking to the same locus.^7^

The mature corneal endothelium consists of a monolayer of regular hexagonal cells that function both as a barrier and as a pump to maintain corneal deturgescence. Endothelial cells have limited proliferative capacity, and cells are lost at a rate of 0.6% per year, resulting in decreasing cell density.^43^, ^44^ Although PPCD1 primarily affects corneal endothelial cells, we were unable to detect *OVOL2* expression in any adult corneal-endothelial-derived tissue or cell line tested. *OVOL2* plays an important role during development, and *Ovol2*^−/−^ mice die at embryonic day 10.5 (corresponding with approximately day 28 of human embryonic development). Prior to this, they fail to develop a precursor of the corneal ectoderm (the optic eminence) and demonstrate impaired neural crest cell survival and migration.^45^ In the developing human embryo, the corneal endothelium is derived from neural crest cells that are located at the boundary of the neural plate and surface ectoderm.^46^ These neural crest cells undergo EMT and migrate to developing tissues, including the anterior segment, where they differentiate to form the corneal endothelial monolayer by the eighth week of gestation.^47^, ^48^ Publically available RNA-seq data derived from 16- to 18-week-old human fetal corneal endothelial samples are also negative for *OVOL2* expression.^34^

We have demonstrated that the mutations identified in the *OVOL2* promoter dysregulate *OVOL2* expression, and we suggest that this will affect the function of downstream genes and pathways, including potential transcriptional regulation of the PPCD3-associated gene *ZEB1*. Our data imply that upregulation of *OVOL2* expression, by the introduction of functional cryptic *cis*-acting transcription factor binding sites, is the disease mechanism. We suggest that the mutations induce inappropriate ectopic expression in the corneal endothelium; however, it is currently not clear whether this occurs during development or in the adult endothelium, or indeed both.^45^ It also remains plausible that *OVOL2* is expressed in vivo at developmental stages not currently investigated and that the promoter mutations identified could lead to loss, or downregulation, of *OVOL2* at a crucial time point in eye development. We speculate that the timing and severity of this inappropriate expression will be mutation dependent, given that different transcription factors (activators or repressors) might bind to the cryptic *cis*-acting promoter sequence elements created by the different mutations. In support of this, we observed differences in disease characteristics and severity as a result of the different mutations. At the severe end of the disease spectrum is a congenital disorder that affects corneal endothelial development by leading to a loss of endothelial cells (family BR1), whereas in the milder Czech families, focal multilayering of endothelial cells^49^ (Figure 2H) and cellular “epithelialization” with increased expression of several epithelial keratins, predominantly KRT7 and KRT19, are characteristic of the disease.^30^ Additionally, malformation of the anterior segment is associated with two of the four mutations identified (families BR2 and BR3).

Aberrant *OVOL2* expression levels are likely to have a range of downstream consequences; however, given that it is a known direct repressor of *ZEB1* (an established PPCD-associated gene), it seems plausible that dysregulation of this OVOL2-ZEB1 regulatory feedback loop is most likely relevant to the mechanism of pathogenesis.^33^ Increased transcriptional repression of *ZEB1* expression due to overexpression of *OVOL2* would be expected to have a similar outcome on the variety of reported PPCD3-associated nonsense, frameshift, and deletion mutations that result in *ZEB1* haploinsufficiency.^10^, ^37^

To address the potential disease mechanism, or mechanisms, leading to the range of corneal endothelial dystrophy phenotypes observed as a consequence of *OVOL2* promoter mutations, it will be important to accurately model corneal disease in an appropriate cell or animal model. Identification of the diseased cell populations inappropriately expressing *OVOL2*, and transcription factors that bind as a result of the mutations, most likely in a cell-dependent context, will be an important next step toward understanding the perturbed regulatory pathways leading to disease.

## Figures and Tables

**Table 1:** Rare Heterozygous Variants within the Refined Locus, Chr20: 17,641,482–18,949,130, Identified by WGS in Family BR1

**Variant No.**	**Coordinates (hg38)**	**Reference Allele**	**Observed Allele**	**Closest Transcript**	**Location**	**dbSNP**	**Allele Count / Total Alleles Screened**				
**1000G**	**UK10K**	**GoNL**	**ExAC**	**UCL WGS**
1	18,057,973	A	ACCGGTTCCGGCGGCCGGGGCTG	*OVOL2*	promotor	**–**	NI	NI	NI	NI	NI
2	18,112,700	T	A	*PET117*	intergenic	rs556855465	2/5,008	4/7,562	NI	NI	NI
3	18,114,422	A	G	*PET117*	intergenic	rs560139714	NI	NI	1/998	NI	NI
4	18,119,439	A	G	*PET117*	intergenic	rs563340932	NI	NI	1/998	NI	NI
5	18,124,502	TAGA	T	*PET117*	intergenic	–	NI	NI	NI	NI	NI
6	18,189,275	CT	C	*CSRP2BP*	intergenic	–	NI	1/7,562	NI	NI	NI
7	18,189,278	C	T	*CSRP2BP*	intergenic	rs772649261	NI	NI	NI	NI	NI
8	18,240,797	G	A	*CSRP2BP*	intergenic	rs552441504	5/5,008	NI	7/998	NI	NI
9	18,273,229	G	A	*ZNF133*	intergenic	rs184537558	5/5,008	NI	NI	NI	NI
10	18,292,765	C	T	*ZNF133*	deep intronic	rs542530373	NI	NI	1/998	NI	NI
11	18,319,195	A	G	*ZNF133*	intergenic	rs530751423	NI	NI	1/998	NI	NI
12	18,379,703	G	A	*LINC00851*	deep intronic	rs558852368	2/5,008	NI	NI	NI	NI
13	18,396,543	T	G	*DZANK1*	missense	rs560809093	NI	NI	1/998	5/120,650	NI
14	18,476,283	G	A	*POLR3F*	deep intronic	rs537806334	NI	NI	1/998	NI	NI
15	18,646,498	G	A	*DTD1*	deep intronic	rs532426738	NI	NI	1/998	NI	NI
16	18,784,373	C	A	*DTD1*	intergenic	rs570664591	NI	NI	1/998	NI	NI
17	18,810,051	G	A	*C20orf78*	non-coding transcript	rs543631581	NI	NI	1/998	NI	NI
18	18,818,189	T	C	*C20orf78*	deep intronic	rs566681725	NI	NI	1/998	NI	NI
19	18,851,016	C	T	*C20orf78*	intergenic	rs550023958	NI	NI	1/998	NI	NI

WGS datasets generated for affected individuals VII:3 and VI:24 were filtered for removal of (1) all variants located outside the refined locus, (2) all variants with a MAF > 0.5% in the publically available 1000G, UK10K, GoNL, and ExAC datasets and in a UCL cohort of 100 WGS datasets (UCL WGS), and (3) variants present in unaffected family member VI:22 and a further eight unrelated and unaffected WGS samples that were sequenced at the same time. Abbreviations are as follows: 1000G, 1000 Genomes Project; deep intronic, more than 100 bp from a defined intron-exon boundary; ExAC, Exome Aggregation Consortium; GoNL, Genomes of the Netherlands; and NI, not identified.

**Table 2:** Rare Heterozygous Variants within the Linked PPCD1 Region, Chr20: 17,335,789–19,665,902, Identified by Targeted Re-sequencing and WGS in Individual V:11 from Family C2

**Variant No.**	**Coordinates (hg38)**	**Reference Allele**	**Detected via Targeted Re-sequencing**	**Detected via WGS**	**Observed Allele**	**Closest Transcript**	**Location**	**dbSNP**	**Allele Count / Total Alleles Screened**				
**1000G**	**UK10K**	**GoNL**	**ExAC**	**UCL WGS**
1	17,779,178	T	not covered	yes	C	*BANF2*	Intergenic	–	NI	NI	NI	NI	NI
2	17,962,621	T	yes	yes	TTCGGGGGAGGGGGG	*SNX5*	deep intronic	–	NI	NI	NI	NI	NI
3	18,010,425	T	not covered	yes	C	*OVOL2*	intergenic	rs62206463	NI	NI	NI	NI	NI
4	18,058,004	A	yes	yes	G	*OVOL2*	promoter	–	NI	NI	NI	NI	NI
5	18,373,822	G	yes	yes	A	*LINC00851*	intergenic	–	NI	NI	NI	NI	NI
6	18,504,053	T	yes	yes	G	*SEC23B*	intergenic	–	NI	NI	NI	NI	NI
7	18,800,653	T	yes	yes	C	*NR_026885*	intergenic	–	NI	NI	NI	NI	NI
8	18,831,605	T	yes	yes	C	*C20orf78*	intergenic	–	NI	NI	NI	NI	NI
9	18,836,917	A	yes	yes	G	*C20orf78*	intergenic	–	NI	NI	NI	NI	NI
10	18,863,686	G	not covered	yes	C	*C20orf78*	intergenic	rs148906570	10/5,008	NI	NI	NI	NI
11	18,870,484	C	not covered	yes	G	*C20orf78*	intergenic	rs150426313	10/5,008	60/7,562	9/998	NI	NI
12	18,937,206	C	yes	yes	G	*C20orf78*	intergenic	–	NI	NI	NI	NI	NI
13	18,950,428	C	not covered	yes	T	*C20orf78*	intergenic	–	NI	NI	NI	NI	NI
14	19,140,530	G	yes	yes	A	*SLC24A3*	intergenic	–	NI	NI	NI	NI	NI
15	19,325,002	A	yes	yes	G	*SLC24A3*	deep intronic	–	NI	NI	NI	NI	NI
16	19,331,987	C	yes	yes	T	*SLC24A3*	deep intronic	rs537549121	9/5,008	2/7,562	NI	NI	NI
17	19,601,355	C	yes	yes	A	*SLC24A3*	deep intronic	–	NI	NI	NI	NI	NI
18	19,637,683	A	yes	yes	G	*SLC24A3*	deep intronic	–	NI	NI	NI	NI	NI

Next-generation sequencing datasets generated for affected individual V:11 were filtered for removal of (1) all variants located outside the refined locus, (2) all variants with a MAF > 0.5% in the publically available 1000G, UK10K, GoNL, and ExAC datasets and in a UCL cohort of 100 WGS datasets (UCL WGS), (3) all variants present in unaffected family member V:10 and identified by targeted re-sequencing, (4) variants present in eight unrelated and unaffected WGS samples that were sequenced at the same time, and (5) variants present in the unaffected (married-in) father (IV:17) and identified by direct sequencing. Abbreviations are as follows: 1000G, 1000 Genomes Project; deep intronic, more than 100 bp from a defined intron-exon boundary; ExAC, Exome Aggregation Consortium; GoNL, Genomes of the Netherlands; and NI, not identified.

**Table 3:** In Silico Analysis of Disease-Associated Variants in the *OVOL2* Promoter

**TF**	**Predicted Effect of *OVOL2* Promoter Variants on TF Binding**				**Relative Abundance of Transcripts, Encoding the TF of Interest, Detected in the Corneal Endothelium by RNA-Seq (RPKM)**	
**c.−339_361dup (Family BR1)**	**c. −370T>C (Families C1–C14, C25, and C30)**	**c. −274T>G (Family BR2)**	**c. −307T>C (Family BR3)**	**Adult**	**Fetal**
ELK1	TF site gained^a^	TF site gained^a^^,^^b^	TF site gained^a^	–	14.01	17.42
GRHL1	TF site gained^a^	–	–	–	0.98	0.04
SLC2A4RG	TF site gained^a^	TF site gained^a^	–	–	11.36^c^	20.46^c^
FLI1	TF site gained^a^	–	–	–	7.85	1.79
ZNF239	–	TF site gained^a^	–	–	2.18	4.30
REL	–	TF site gained^a^	–	–	2.17	1.00
ZNF143	–	–	TF site lost^a^	–	12.06	7.54
DMRTA2	–	–	–	TF site gained^a^	0.65	0.02
RABL6	–	–	–	TF site gained^a^	14.98^c^	10.10^c^
RFX3	–	–	–	TF site gained^a^	2.39	5.07
T	–	–	–	TF site lost^a^	0.00	0.01
SP1	TF site gained^b^	TF site gained^b^	–	–	12.52	17.53
EGR1	TF site gained^b^	–	–	–	134.09	84.08
ATF2	–	–	–	TF site gained^b^	11.78	13.26
SRF	–	–	TF site gained^b^	–	21.42	16.48
ETS1	–	–	TF site lost^b^	–	10.92	9.01

*OVOL2* variants are annotated in accordance with GenBank: NM_021220, and +1 represents the start of translation. Human corneal endothelial RNA-seq reads for three adult and two fetal (16- to 18-week-old) samples (study SRP01140 from ArrayExpress) were aligned to the human reference genome (NCBI Genome build 38; GCA_000001405.15_GRCh38 without alternate contigs) with STAR v.2.5.0. Abbreviations are as follows: RKPM, reads per kilobase per million mapped reads; and TF, transcription factor.

a MatInspector, which is a software tool that utilizes a large library of matrix descriptions for transcription factor binding sites to locate matches in DNA sequences.

b AliBaba 2.1, which is a program that predicts transcription factor binding sites in an unknown DNA sequence by utilizing binding sites collected in the public database TRANSFAC.

c RKPM values also encompass flanking transcripts.

## Web Resources

The URLs for data presented herein are as follows:

1000 Genomes, www.1000genomes.org
Alibaba 2.1, http://www.gene-regulation.com/pub/programs/alibaba2/index.html
ANNOVAR, http://www.openbioinformatics.org/annovar/
ClustalW2, http://www.ebi.ac.uk/Tools/msa/clustalw2/
dbSNP, http://www.ncbi.nlm.nih.gov/projects/SNP/
ENCODE, https://genome.ucsc.edu/ENCODE/
Ensembl, http://www.ensembl.org/index.html
Exome Aggregation Consortium (ExAC) Browser, http://exac.broadinstitute.org/
ExomeDepth, http://cran.r-project.org/web/packages/ExomeDepth/index.html
Genome Analysis Toolkit (GATK), www.broadinstitute.org/gatk/
Genomes of the Netherlands (GoNL), http://www.nlgenome.nl/
Interactive Genomics Viewer (IGV), http://www.broadinstitute.org/software/igv/
MatInspector, https://www.genomatix.de/online_help/help_matinspector/matinspector_help.htmlOnline
NCBI Genome build GRCh38, ftp://ftp.ncbi.nlm.nih.gov/genomes/all/GCA_000001405.15_GRCh38/seqs_for_alignment_pipelines.ucsc_ids/
NHLBI Exome Sequencing Project (ESP) Exome Variant Server, http://evs.gs.washington.edu/EVS/
Novoalign, http://www.novocraft.com/products/novoalign/
OMIM, http://www.omim.org
PolyPhen-2, http://genetics.bwh.harvard.edu/pph2/
RefSeq, http://www.ncbi.nlm.nih.gov/refseq/
SAMtools, http://www.htslib.org/
SeattleSeq Annotation, http://snp.gs.washington.edu/SeattleSeqAnnotation141/
SIFT, http://sift.jcvi.org/
UCSC Genome Browser, https://genome.ucsc.edu/
UK10K, http://www.uk10k.org/
Variant Effect Predictor, http://www.ensembl.org/info/docs/tools/vep/index.html

## Supplemental Data

Document S1. Figure S1 and Tables S1 and S2

Document S2. Article plus Supplemental Data
