# Supplementary material for: Autosomal-Dominant Corneal Endothelial Dystrophies CHED1 and PPCD1 Are Allelic Disorders Caused by Non-coding Mutations in the Promoter of OVOL2
Source: Am J Hum Genet. 2015 Dec 31;98(1):75–89. doi: 10.1016/j.ajhg.2015.11.018 (PMC4716680; doi:10.1016/j.ajhg.2015.11.018)
Supplement: Document S1. Figure S1 and Tables S1 and S2 [file mmc1.pdf]

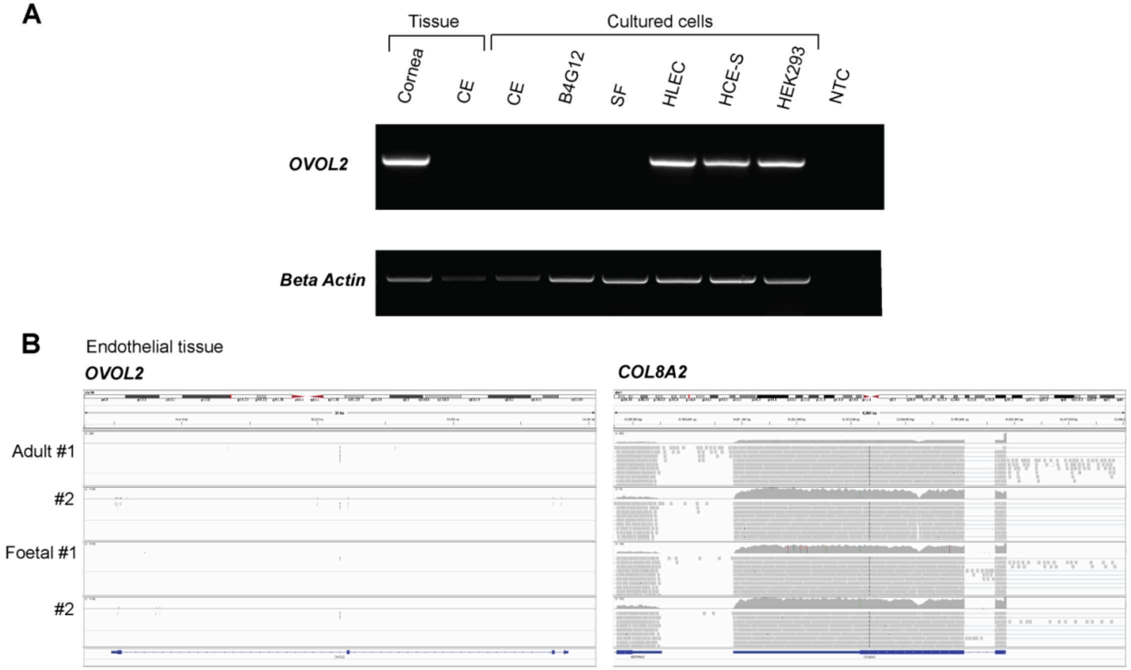

Figure S1: Testing for expression of *OVOL2* in human corneal tissue and various cell types by reverse transcription (RT) PCR and utilisation of RNA sequencing data. (A) RNA was extracted from a variety of tissue and cell types, cDNA was synthesised and RT-PCR reactions were performed using intron spanning *OVOL2* and beta actin specific primers**.** CE = adult corneal endothelium, B4G12 = an immortalised cell line of human corneal endothelial origin [2], SF = stromal fibroblasts, HLEC = human limbal epithelial cells, HCE-S= human corneal epithelial cells (spontaneously immortalised with progenitor-like characteristics) [3], HEK293 = human embryonic kidney derived cell line 293, NTC= no template control. **(B)** Interrogation of RNA-seq data, using Integrated Genomics Viewer, derived from adult and fetal human corneal endothelial samples [4] revealed no evidence of *OVOL2* expression. *COL8A2*, a gene known to be expressed in corneal endothelium, is shown to be highly expressed in this tissue.

Table S1: Refinement of the CHED1 locus in family BR1

Genetic markers	Coordinates Hg19	Coordinates Hg38	V:20	VI:2	VI:7	VI:17	VI:24	VII:3	VII:7	VII:13
rs2072958	20:17,505,226	20:17,524,581	TC	TC	CC	TC	-	CC	TT	TT
*rs6044930	20:17,622,127	20: 17,641,482	TC	CC	TT	TC	-	TC	CC	CC
**rs6136169**	**20:17,679,400**	**20: 17,698,755**	**TC**	**TC**	**TC**	**CC**	**TC**	**CC**	**TC**	**TC**
**rs2296903**	**20:17,705,226**	**20: 17,724,581**	**CC**	**TC**	**TC**	**CC**	**CC**	**CC**	**CC**	**CC**
**rs6080830**	**20:17,771,113**	**20: 17,790,468**	**GA**	**GA**	**GA**	**GA**	**GA**	**GA**	**GA**	**AA**
**D20S182**	**20:17,834,284-17,834,622**	**20:17,858,720-17,858,918**	**11,12**	**16,12**	**17,12**	**16,12**	**11,12**	**11,12**	**14,12**	**16,12**
**D20S885**	**20:17,958,679**	**20:17,983,211-17,983,337**	**16,17**	**18,17**	**16,17**	***,17**	***,17**	**16,17**	**18,17**	***,17**
**D20S475**	**20:18112800-18113074**	**20:18,137,213-18,137,354**	**7,8**	**8,8**	**7,8**	**8,8**	**7,8**	**7,8**	**7,8**	**7,8**
**D20S860**	**18,329,131**	**20:18,353,530-18,353,767**	**17,15**	**18,15**	**16,15**	**17,15**	**11,15**	**14,15**	**11,15**	**16,15**
**rs6081232**	**20:18,571,572**	**20:18,590,928**	**GA**	**AA**	**GA**	**AA**	**-**	**GA**	**GA**	**GA**
**rs6136525**	**20:18,806,046**	**20:18,825,402**	**TT**	**TT**	**TT**	**CT**	**CT**	**CT**	**CT**	**TT**
*rs6105994	20:18,929,774	20:18,949,130	-	-	AA	-	-	-	-	GG
rs10485587	20:18,950,921	20:18,970,277	-	-	GG	-	-	-	-	AA
rs1535019	20:19,427,807	20:19,447,163	-	-	GG	-	-	-	-	CC

Fourteen genetic markers on chromosome 20p, located within the linkage region originally reported by Toma *et al.* [1] were genotyped in 8 affected members of Family BR1. Alleles segregating with disease are highlighted in bold. Alleles denoted with * represent cases where the calling of the second copy was inconclusive. Alleles segregating with disease are highlighted in red.

Table S2: Shared rare heterozygous variants identified by whole exome sequencing (WES) in affected members of family BR1 (VII:23 and VI:6). WES data sets were filtered to remove: **(1)** all variants not present in both individuals VI:5 and VII:13 **(2)** all variants not present in the heterozygous state **(3)** all variants with a minor allele frequency (MAF) >0.5% in the 1KG dataset, ESP dataset and in our UCL whole exome sequence (UCL WES) control datasets.

**Location (Hg38)**	**Nucleotide change**	**Protein change**	**SIFT (tolerance index 0-1)**	**Polyphen 2 (HumVar score 0-1)**	**Blosum 62 score (-4 to 11)**	**Minor allele frequency (MAF) in control datasets**	
						**ExAC allele number (Eurpoean alleles)**	**UCL WES**
Chr20:18,396,543	c.1540A>C	p.(Ile514Leu)	0.33 (Tolerated)	0.015 (Benign)	2	5/120,650 (5/66,682)	0/4,334

In silico analysis of rare variants identified is presented**.** SIFT results are reported to be tolerant if tolerance index is ≥0.05 or intolerant if tolerance index is <0.05. Polyphen 2 appraises mutations quantitatively as benign, possibly damaging (POS) or probably damaging (PRD) based on the model's false positive ratio. HumVar score is the preferred model for diagnostics of Mendelian diseases which requires distinguishing mutations with drastic effects from all the remaining human variation, including abundant mildly deleterious alleles. Blosum62 substitution matrix score positive numbers indicate a substitution more likely to be tolerated evolutionarily and negative numbers suggest the opposite. The cDNA is numbered according to transcript ID ENST00000262547 (*DZANK1*). ExAC denotes Exome Aggregation Consortium (ExAC Browser),http://exac.broadinstitute.org/ (accessed July 2015). UCL WES refers to 4,334 WES datasets or individuals of varying ethnicity.

References

1. Toma, N.M., Ebenezer, N.D., Inglehearn, C.F., Plant, C., Ficker, L.A., and Bhattacharya, S.S. (1995). Linkage of congenital hereditary endothelial dystrophy to chromosome 20. Human molecular genetics *4*, 2395-2398.
2. Valtink, M., Gruschwitz, R., Funk, R.H., and Engelmann, K. (2008). Two clonal cell lines of immortalized human corneal endothelial cells show either differentiated or precursor cell characteristics. Cells, tissues, organs *187*, 286-294.
3. Notara, M., and Daniels, J.T. (2010). Characterisation and functional features of a spontaneously immortalised human corneal epithelial cell line with progenitor-like characteristics. Brain research bulletin *81*, 279-286.
4. Chen, Y., Huang, K., Nakatsu, M.N., Xue, Z., Deng, S.X., and Fan, G. (2013). Identification of novel molecular markers through transcriptomic analysis in human fetal and adult corneal endothelial cells. Human molecular genetics *22*, 1271-1279.
